# Does leaf anatomy aid in species identification of *Butia* (Arecaceae)?

**DOI:** 10.1093/aobpla/ply046

**Published:** 2018-07-28

**Authors:** Bruno Francisco Sant’Anna-Santos, Samuel Alves dos Santos, Elaine L P Nunes, Dayana Maria Teodoro Francino, Wellington Geraldo Oliveira Carvalho Júnior

**Affiliations:** 1 Laboratório de Anatomia e Biomecânica Vegetal, Departamento de Botânica, Setor de Ciências Biológicas, Universidade Federal do Paraná (UFPR), Curitiba, PR, Brazil; 2 Laboratório de Anatomia e Dendrologia, Universidade Federal de Minas Gerais (UFMG), Avenida Universitária, 1000, Bairro Universitário, Montes Claros, MG, Brazil; 3 Departamento de Ciências Biológicas, Universidade Federal dos Vales do Jequitinhonha e Mucuri (UFVJM), Rodovia BR 367, Diamantina, MG, Brazil

**Keywords:** Attaleineae, leaf anatomy, Palmae

## Abstract

*Butia* is a neotropical genus whose identification is based mostly on characters from external morphology, which are sometimes variable or inadequate for species differentiation. We aimed to verify if leaf anatomy of 18 *Butia* species brings new characters suitable for species identification and if it corroborates the phylogenetic relationship within the genus. Moreover, we propose an anatomical key to assist in species identification. Pinnae were collected and subjected to the usual techniques for light and scanning electron microscopies. The anatomical key was created with the aid of Xper^2^ software, based on the importance of characters to distinguish species according to the Jaccard index. All species have isobilateral mirrored mesophyll, amphistomatic leaves and secondary vascular bundles with sclerenchymatic sheath reinforcement connected to the hypodermis. Among the species studied, *B. marmorii* and *B. matogrossensis* showed exclusive characters. For the other species, up to five characters are sufficient for delimitation. Our anatomical key presents relevant characters that allow the identification of the recognized species of *Butia*. Reliable anatomical characters of easy observation, especially the raphides, are valuable in species distinction. Leaf anatomy, already used to support new taxa in related genera like *Allagoptera* and *Syagrus*, can also be useful to validate questionable *Butia* species and differentiate between similar species but do not reflect the proposed relationship between *Butia* species.

## Teaser

Our paper presents reliable anatomical characters of the pinnae that allowed the identification of the recognized species of *Butia* through a purely anatomical key, in which specific identification is performed by a set of up to five characters. Leaf anatomy, already used to support new taxa in related genera, can also be useful to validate questionable *Butia* species and differentiate between similar species, but surprisingly does not reflect the proposed phylogenetic relationship between species of this genus.

## Introduction

In spite of its monophyly (Merrow *et al.* 2009,^2015^), *Butia* is morphologically highly diverse (Glassman 1970; Dransfield *et al.* 2008; Noblick 2010, 2014), causing difficulties in species delimitation (Soares *et al.* 2014) and considerable changes on the taxonomy of the genus. According to Noblick (2014), between 2004 and 2014, the number of accepted species included in the genus has risen from 9 to 24, and more species are expected to be described.

Indeed, there is no consensus regarding the present total number of species, some of them being still questionable according to Noblick (2014). Noblick (2010) reported that 18 species occur naturally, predominantly in areas in southern Brazil, eastern Paraguay, north-eastern Argentina, and north-western and south-eastern Uruguay. Brazil possesses the majority of the existing species of *Butia*, at least 16. Noblick (2010) also recognized 18 species, while Soares (2015) recognized 20 valid species and listed 11 names as synonyms or doubtful species. Presently, the World Checklist of Selected Plant Families—WCSP—(2018) lists 22 species, while the Flora do Brasil 2020 (Heiden *et al.* 2018) recognizes 19 species and two varieties occurring in Brazil.

As an example of the taxonomical instability of *Butia*, we can cite *B. leiospatha*, which is considered a synonym of *B. capitata* in WCSP (2018) and *B. lallemantii* in Flora do Brasil 2020 (Heiden *et al.* 2018). *Butia leiospatha* was originally described by Barbosa Rodrigues as *Cocos leiospatha* in 1877, but, according to Glassman (1970), he did not indicate any specimen or type and the description did not fit the illustration of it. On the *B. leiospatha* description, plants are usually acaulescent but, the illustration depicts are relatively tall tree. Drude (1881) described *C. leiospatha* var. *angustifolia* based on a specimen collected by Warming in 1845 in Lagoa Santa municipality, Minas Gerais State, Brazil. Later, Beccari (1916) elevated *Butia* to genus and considered *C. leiospatha* var. *angustifolia* as a synonym of *Butia bonneti.*Glassman (1970), on the other hand, considered the specimen collected by Warming unsuitable for a correct identification due to the lack of data and poor vouchering; he suggested that this specimen could represent a young state of *B. capitata* or belong to *B. arenicola.* Also, he considered *B. bonnetii*, *B. poni* and *B. pungens* as doubtful. Characters derived from the external morphology such as the general aspect of the plant, size of inflorescences and fruit colour may be highly variable intraspecifically (Noblick 2014). This variability put together with the relevance attributed to a given character by each author when circumscribing a taxon, perpetuate the taxonomic deadlocks regarding the genus.

Currently, there are two identification keys proposed by Noblick (2010) and Soares *et al.* (2014)—modified by Noblick (2014); both are based solely on external morphology. The most comprehensive key includes 17 species (Noblick 2014) is only effective, according to the author, if used with mature plants. Immature specimens of *Butia* are difficult to discriminate of due to the influence of their age upon plant and inflorescence sizes. Since part of those characters are naturally variable or may vary according to plant age (Noblick 2014) and environment (Noblick 2010), using only them might limit identification or even induce error. Thus, it is imperative to find new characters suitable for improving *Butia* identification.

Because of this, taxonomy of Arecaceae traditionally has also been based on anatomical aspects of the organs, mainly of the leaf blade (Tomnlinson 1961; Tomnlinson *et al.* 2011; Noblick 2013, 2014; Martins *et al.* 2015; Sant’Anna-Santos *et al.* 2015; Pinedo *et al.* 2016; Vianna *et al.* 2017). The leaf blade has provided useful characters to discriminate between species in some genera of the family, such as *Oenocarpus* (Silva and Potiguara 2008; Tomnlinson *et al.* 2011), *Syagrus* (Glassman 1970; Tomnlinson *et al.* 2011; Noblick 2013; Noblick *et al.* 2014), *Allagoptera* (Tomnlinson *et al.* 2011; Martins *et al.* 2015; Pinedo *et al.* 2016), *Parajubaea* (Meerow *et al.* 2009; Tomnlinson *et al.* 2011), *Acrocomia* (Vianna *et al.* 2017) and even *Butia* (Noblick 2014; Sant’Anna-Santos *et al.* 2015). A detailed comparative study of the leaf anatomy of *Butia* provided useful characters that corroborated the split of *B. odorata* from *B. capitata* and aid in their distinction (Sant’Anna-Santos *et al.* 2015).

However, there are more comprehensive anatomical treatments for other genera of Attalaineae subtribe. Noblick (2013) proposed an identification key for 26 acaulous species of *Syagrus*, based exclusively on anatomical characters of the leaf and verified that leaf anatomy corroborates their phylogenetic relationship. Therefore, this study aimed to verify if leaf anatomy of *Butia* species brings new characters suitable for species identification and propose an anatomical key. Moreover, we aimed to verify if, as for other Attaleinae genera, leaf anatomy can corroborate the phylogenetic relationship within *Butia* too. We analysed all the accepted by Noblick (2014) plus one specimen of *B. leiospatha* currently synonymized under *B. capitata*.

## Materials and Methods

Samples of *Butia archeri*, *B. campicola*, *B. capitata*, *B. catarinensis*, *B. eriospatha*, *B. exospadix*, *B. lallemantii*, *B. leiospatha*, *B. leptospatha*, *B. lepidotispatha*, *B. marmorii*, *B. matogrossensis*, *B. microspadix*, *B. paraguayensis*, *B. pubispatha*, *B. purpurascens* e *B. yatay* were collected from specimens cultivated in the Botanic Garden of the Plantarum Institute, in Nova Odessa municipality, São Paulo State, Brazil. Vouchers were deposited in the herbarium of the Botanic Garden Plantarum under the numbers HPL-1531, HPL-11480, HPL-10332, HPL-11412, HPL-13194, HPL-11513, HPL-11477, HPL-3405, HPL-11479, HPL-11476, HPL-11494, HPL-10265, HPL-11628, HPL-13195, HPL-11475, HPL-13196, HPL-7659. For each species, at least three specimens were analysed, except for *B. leiospatha* that was collected from a single specimen. Samples of *B. odorata*, *B. capitata* and *B. archeri* were collected in the field (in Tapes municipality, Rio Grande do Sul; in Lontra municipality, Minas Gerais and Diamantina municipality-Type locality, Minas Gerais, respectively). These vouchers were deposited, respectively, in the Alarich Schultz Herbarium of the Museum of Natural Sciences, Rio Grande do Sul Zoobotanical Foundation (HAS-47695), in the Herbarium of the Department of Botany, Federal University of Minas Gerais (BHCB-144649) and in Herbarium of Federal University of Jequitinhonha and Mucuri Valleys (DIAM-3157). Herbarium acronyms are according to Thiers (2018, continuously updated).

Samples for epidermic dissociations were fixed in 50 % ethanol, while those for semi-permanent slides were fixed in FAA (solution of 47 % formaldehyde, acetic acid and 70 % ethanol, 1:1:18 by volume) (Johansen 1940), and those for scanning electron microscopy analysis were fixed in Karnovsky’s solution (Karnovsky 1965).

For the light microscopy studies, samples were previously softened for 12 h in a solution of 10 % ethylenediamine, as described by Sant’Anna-Santos *et al.* (2015). Thus, samples were sectioned on a microtome table (model LPC, Rolemberg e Bhering Comércio e Importação LTDA., Belo Horizonte, Brazil) with a disposable razor blade. Both sections and epidermal fragments were stained with 1 % Safranin and 1 % Astra blue, mounted between slide and coverslip with distilled water for photo documentation and, subsequently, with glycerin water (Bukatsch 1972, modified).

Pinnae fragments with ~1 cm^2^ were dissociated in a solution of 10 % nitric acid and 10 % chromic acid 10 % (v/v) (Jensen 1962) for analysis of the epidermis in frontal view. The resulting epidermal fragments were stained with 1 % Safranin and 1 % Astra blue.

Fixed samples were dehydrated in an ethylic series (30, 50, 70, 90 and 100 %) and embedded in methacrylate (Historesin, Leica Instruments, Heidelberg, Germany). Sections with 5 μm were obtained through a manual rotary microtome Reichert and stained with toluidine blue O pH 4.0 (O’Brien and McCully 1981). The slides were mounted using water for observation and documentation.

For morphological analysis of epicuticular waxes, fragments of the median portion of pinnae (0.5 cm^2^) were dehydrated in ethylic series and dried by critical point drier (Balzers CPD 030) and covered with gold in a Sputter Coater (Balzers SCD 050). For analysis of silica bodies, part of the samples was transferred from the fixative solution to a solution of 30 % glycerol for 3 h. Then, they were transferred to liquid nitrogen for 30 s and cryofractured. Afterwards, they were dehydrated in a graded series of propanone, critical-point dried and sputter-coated with gold.

Observation and photo documentation were performed under a light microscope (Primo Star, Zeiss, Berlin, Germany) with a coupled digital camera (AxioCam ERc5s, Zeiss, Berlin, Germany). After the processing of the samples, images were obtained in a scanning electron microscope (JSMT200, Jeol Co., Tokyo, Japan).

Thirty-two qualitative anatomical characters considered reliable (without variation between specimens of the same species) were selected to the analysis of the phenotypic similarity between species. The resulting matrix scoring presence and absence of characters was used to perform a cluster analysis at PCCORD 5.0 software. The same characters were also used to elaborate a dichotomous key (software Xper^2^, version 2.3.1) based on the importance of characters to differentiate species according to Jaccard index (Mueller-Dombois and Ellemberg 1974).

## Results

**Figure 1.**
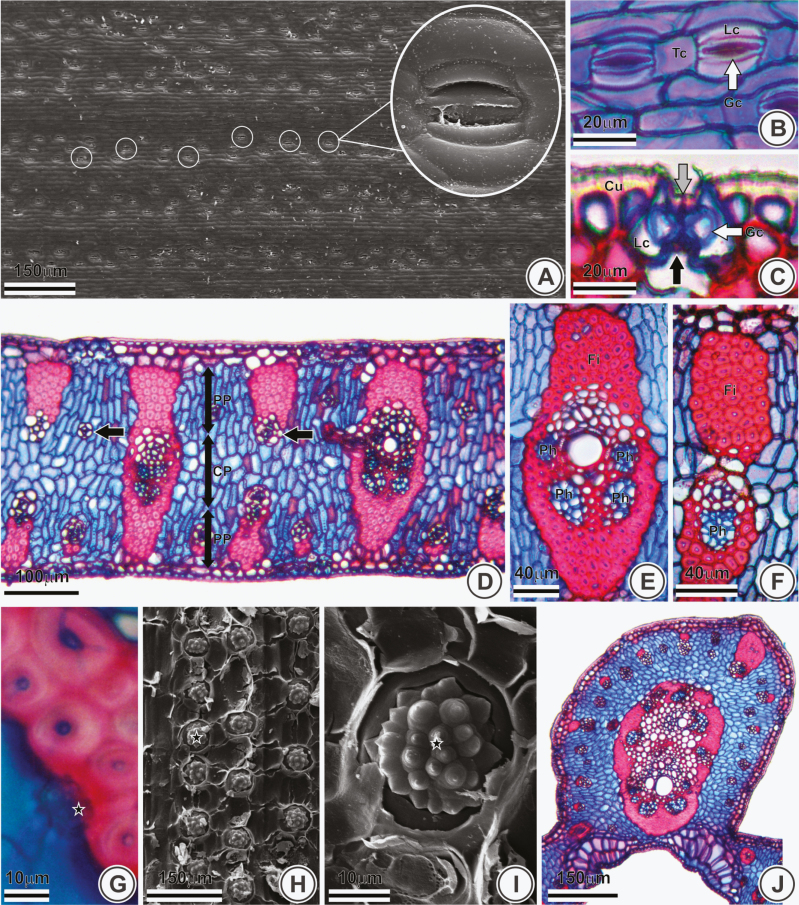
Epidermis and mesophyll of *Butia pubispatha* (A, E and J), *B. campicola* (B), *B. microspadix* (C and D), *B. catarinensis* (F), *B. odorata* (G) and *B. capitata* (H and I). Front view (A and B) and transverse sections (C–G and J) under light microscopy and scanning electron microscopy (H and I). (A) Stomata organized in rows (white circles). (B) Tetracytic stomata. (C) Inner (black arrow) and outer (grey arrow) stomatal ledge. (D) Isobilateral mesophyll and type 3 vascular bundle (black arrows). (E and F) Vascular bundles: type 1 (E) and 2 (F). (G–I) Silica body (stars). (J) Midrib. TC, terminal cell; LC, lateral cell; GC (white arrows), guard cell; Cu, cuticle; PH, phloem; PP, palisade parenchyma; CP, central parenchyma; Fi, fibres.

Stomata are organized longitudinally and parallel in rows (Fig. 1A), tetracytic and composed of elongated lateral subsidiary cells (Fig. 1B)—transversely arciform (Fig. 1C)—and hexagonal terminal (polar) subsidiary cells (Fig. 1B). The guard cells show prominent outer and inner stomatal ledge (Fig. 1C). Ordinary cells of the epidermis possess anticlinal walls with a straight outline (Fig. 1B). The epidermis is covered by a thick cuticle (Fig. 1C).

Pinnae are amphistomatic (Fig. 1D). The mesophyll is isobilateral, compact and with one band of central chlorenchyma of bulky elongate-spherical cells that lies in between two bands of palisade parenchyma (Fig. 1D). Adjacent to the epidermis, on both sides of the pinnae, one or two rows of hypodermis are present (Fig. 1D), whose cells are larger than the ordinary epidermal ones.

The secondary and tertiary vascular bundles of *Butia* can mostly be classified into three types. Type 1 includes bundles of larger calibre, with four poles of sieve elements plus companion cells and a sclerenchymatic sheath extension associated to adaxial and abaxial hypodermis (Fig. 1D and E). Type 2 includes bundles similar to type 1, but with undivided phloem (Fig. 1D and F). Type 3, the most frequent, includes bundles with smaller calibre and with a sclerenchymatic sheath extension associated only to the hypodermis of one side; they usually occur above or below of a similar bundle or, less frequently, above or below a fibre cap or a vascular tissue not surrounded by a sclerenchymatic sheath (Fig. 1D). The stegmata cells, with druse-like silica bodies, occur in association with the fibres of the sclerenchymatic bundle sheath (Fig. 1G–I). The midrib is more protruding on the adaxial than on the abaxial surface (Fig. 1J) in all studied species.

**Figure 2.**
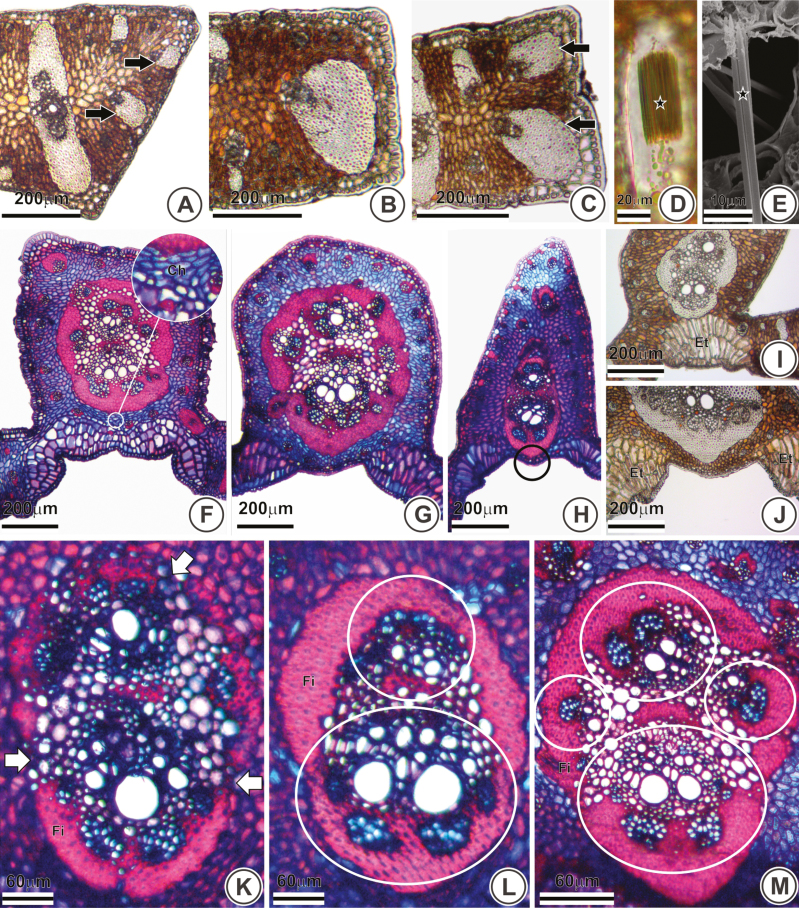
Leaf anatomy of *Butia leptospatha* (A), *B. matogrossensis* (B and K), *B. paraguayensis* (C), *B. exospadix* (D and I), *B. capitata* (E), *B. catarinensis* (F), *B. purpurascens* (G), *B. marmorii* (H), *B. archeri* (J), *B. lallemantii* (L) and *B. yatay* (M) under light (A–D and F–M) and scanning electron microscopy (E). Transversal (A–C, E, F–M) and longitudinal sections (D). (A–C) Leaf margin with reinforced vascular bundles (black arrows). (D–E) Raphides (stars). (F) Chlorenchyma (white circle). (G) Round midrib. (H) Slightly protruding midrib (black circle). (I–J) Expansion tissue. (K) Discontinuous fibre ring (white arrow). (L–M) Vascular bundles (white circles). Ch, chlorenchyma; ET, expansion tissue; Fi, fibres.

In transverse sections, the margin can be either deltoid (Fig. 2A) or quadrangular (Fig. 2B and C). However, some species (*B. capitata*, *B. microspadix*, *B. lallemantii* and *B. pubispatha*) showed some specimens with pinnae with deltoid margins and other with quadrangular margins, demonstrating that this is not a reliable character to distinguish these species.

Two patterns of sheath reinforcement were observed in tertiary bundles of the margin. The first, and least frequent, contains a single reinforced vascular bundle (Fig. 2B), the second, and more frequent, contains two (Fig. 2C), being observed in 11 species. Crystal idioblasts containing raphides (Fig. 2D and E) occur within the margins and intermediary region of pinnae in eight species.

In transverse sections, the midrib was truncate (Fig. 2F), round (Fig. 2G) or triangular (Fig. 2H); the last one was exclusive of *B. marmorii*. The expansion tissue was either continuous (Fig. 2I)—the two caps are connected—or discontinuous (Fig. 2J)—the two caps are separated. The discontinuous expansion tissue was observed in 10 out of 18 species. Regardless of the continuity of the expansion tissue, in 12 species, it was three-layered (Fig. 2G), sometimes with four or more layers (Fig. 2F) or two-layered (Fig. 2H).

The fibrous ring that surrounds the vascular system of the midrib is connected to the abaxial hypodermis (Fig. 2H) in four species. Nonetheless, most species possess at least one layer of chlorenchyma between the fibrous ring and the hypodermis (Fig. 2F and G). The midrib is slightly protruding from the abaxial surface of four species (Fig. 2H). The fibrous ring is discontinuous only in *B. matogrossensis* (Fig. 2K), while in the remaining species, it is continuous, as observed in *B. lallemantii* and *B. yatay* (Fig. 2L and M, respectively). In seven species, the fibrous ring is protruding in the abaxial side (Fig. 2J), a feature that is absent in the remaining species, as seen in *B. catarinensis* (Fig. 2F) for example. Six species possess the vascular system composed of two collateral bundles (Fig. 2L), while the remaining possess three or more collateral bundles (Fig. 2M).

**Figure 3.**
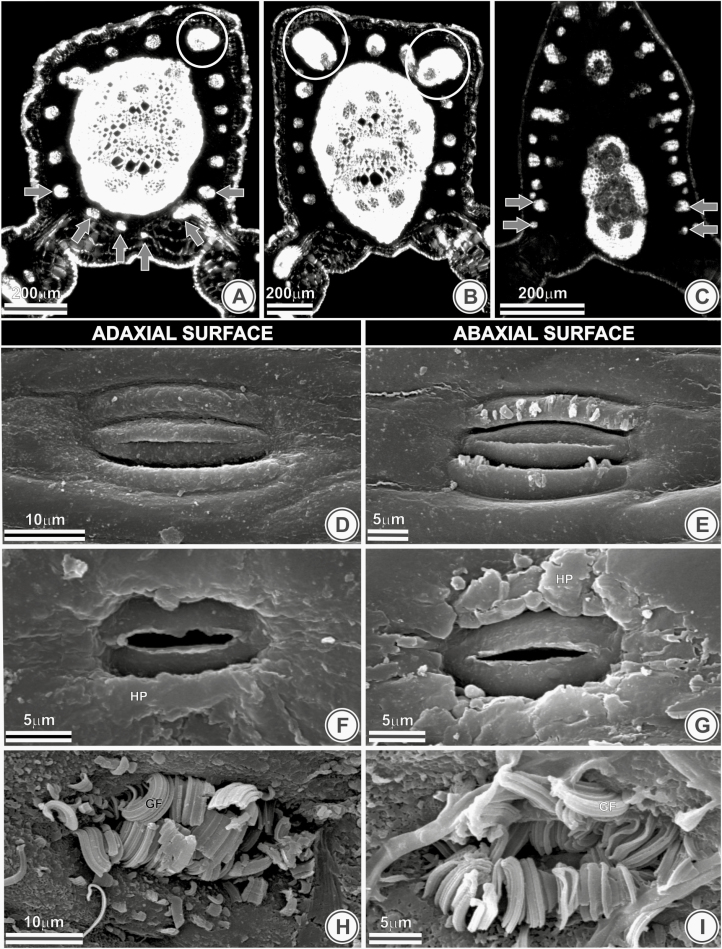
(A) Accessory vascular bundles (A–C) and epicuticular waxes types (D–I) of *Butia catarinensis* (A), *B. yatay* (B), *B. marmorii* (C), *B. leptospatha* (D and E), *B. microspadix* (F and G) and *B. pubispatha* (H and I). (A) Accessory vascular bundles surround the fibrous central ring completely (grey arrow) and a single vascular bundle with sheath reinforcement (white circle). (B) Two bundles with a sheath reinforcement (white circles). (C) Accessory bundles with the same size. (D and E) No conspicuous deposition of epicuticular waxes. (F and G) Horizontal plates (HP) of epicuticular waxes. (H and I) Horizontal plates of epicuticular waxes associated with filaments hook-shaped (GF).

The accessory vascular bundles surround the fibrous central ring completely in *B. catarinensis* (Fig. 3A) and *B. odorata*. These accessory bundles also showed variation in number and size of sclerenchymatic sheath reinforcement. Regarding their number, five classes (in series of five bundles) are being proposed here. Class I (two to seven bundles) occurs only in *B. eriospatha*, while Class II (8 to 13 bundles) occurs in three species. class III (14 to 19 bundles) was observed in six species, while Class IV (20 to 25 bundles) was observed in six species. class V (26 to 31 bundles) was observed in *B. marmorii* only (Fig. 3C). Three types of sheath reinforcement were observed concerning its size. Species with type 1—five of them—contain a single huge vascular bundle in calibre compared to the remainder (Fig. 3A). Most species showed the type 2, in which two huge vascular bundles in calibre compared to the remainder (Fig. 3B). Type 3, whose all accessory bundles are minute and approximately with the same size (without any huge vascular bundle), was observed only in three species (Fig. 3C).

Regarding the epicuticular wax, two species showed no conspicuous deposition (type 1) (Fig. 3D and E). The other species showed conspicuous depositions of wax either in horizontal plates (type 2) (Fig. 3F and G) or associated with filaments hook-shaped (type 3) (Fig. 3H and I). The latter was observed in 13 species.

**Figure 4.**
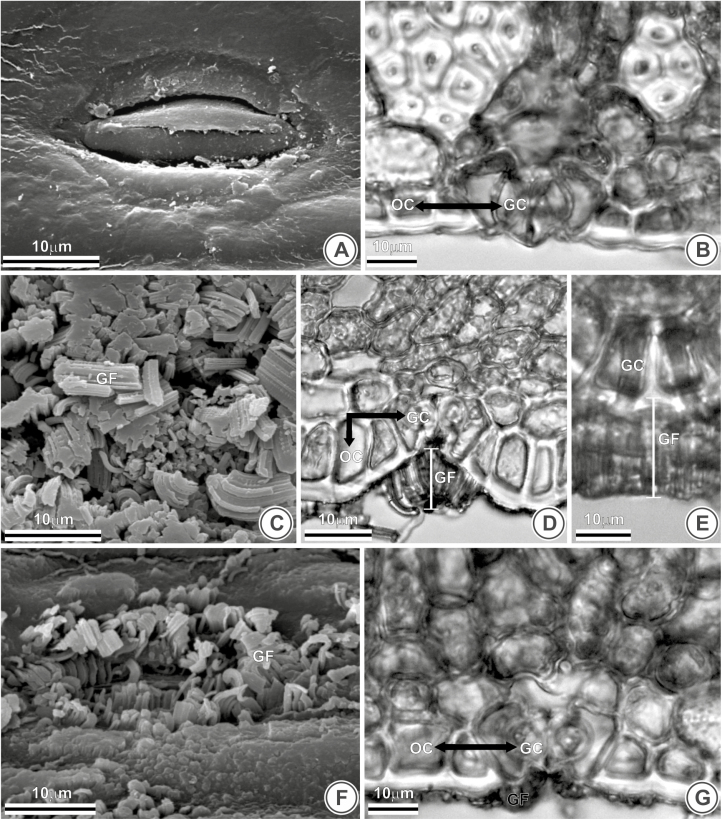
Epicuticular waxes and stomatal position of *Butia exospadix* (A and B), *B. catarinensis* (C–E) and *B. campicola* (F and G) in frontal view (A, C and F), transversal (B, D and G) and longitudinal section (E). (A) Uncoated stomata. (B) Stomata in the same level of ordinary epidermal cells. (C–E) Coated stomata sunken within the epidermis. (F) Coated stomata. (G) Stomata at the level of ordinary cells. HP, horizontal plates; GF, filaments hook-shaped; OC, ordinary cell; GC, guard cell.

Thirteen species have their stomata coated by epicuticular wax, and other four species do not—e.g. *B. exospadix* (Fig. 4A). In those species with uncoated stomata, they are at the same level of ordinary epidermal cells (Fig. 4B), except for *B. eriospatha*. In most species with coated stomata (Fig. 4C–F), they are sunken within the epidermis (Fig. 4D), as seen in *B. catarinensis*; an exception is *B. campicola*, where they are at the level of ordinary cells (Fig. 4F and G).

Thirty-two characters were selected for the multivariate analysis (Table 1). Based on the selected anatomical characters (Table 2), we elaborated this identification key for *Butia*:

**Table 1.** Anatomical characters used for the multivariate analysis.

Characters	Species	*B. archeri*	*B. campicola*	*B. capitata*	*B. catarinensis*	*B. eriospatha*	*B. exospadix*	*B. lallemantii*	*B. leiospatha*	*B. leptospatha*	*B. lepidotispatha*	*B. marmorii*	*B. matogrossensis*	*B. microspadix*	*B. odorata*	*B. paraguayensis*	*B. pubispatha*	*B. purpuracens*	*B. yatay*
PR		0	1	1	1	0	1	0	0	1	0	1	0	1	0	0	1	0	0
ET	C	0	1	1	1	1	1	1	0	1	0	0	0	0	0	0	0	0	0
	I	1	0	0	0	0	0	0	1	0	1	1	1	1	1	1	1	1	1
Midrib shape	Tru	1	1	0	0	1	1	1	1	1	0	0	1	1	1	1	0	0	1
	Rou	0	0	1	1	0	0	0	0	0	1	0	0	0	0	0	1	1	0
	Tri	0	0	0	0	0	0	0	0	0	0	1	0	0	0	0	0	0	0
Stomata position	BL	1	0	1	1	1	0	1	1	0	1	1	1	0	1	1	0	1	1
	SL	0	1	0	0	0	1	0	0	1	0	0	0	1	0	0	1	0	0
ABSCM		0	0	0	1	0	0	0	0	0	0	0	0	0	1	0	0	0	0
FRMH		0	0	0	0	0	1	0	0	0	0	1	0	0	0	1	1	0	0
PCFRH		1	0	1	1	1	0	1	1	1	1	1	1	1	0	1	1	1	1
EW	T1	0	0	0	0	0	1	0	0	1	0	0	0	0	0	0	0	0	0
	T2	0	0	0	0	1	0	0	0	0	0	0	0	1	1	0	0	0	0
	T3	1	1	1	1	0	0	1	1	0	1	1	1	0	0	1	1	1	1
NABMMVS	2–7	0	0	0	0	1	0	0	0	0	0	0	0	0	0	0	0	0	0
	8–13	1	0	1	0	0	0	0	0	0	0	0	0	0	0	1	0	0	0
	14–19	0	0	0	1	0	0	1	1	0	1	0	0	1	0	0	1	0	1
	20–25	0	1	0	0	0	1	0	0	1	0	0	1	0	1	0	0	1	0
	26–31	0	0	0	0	0	0	0	0	0	0	1	0	0	0	0	0	0	0
ETE	2	0	0	0	0	0	0	0	0	0	0	1	0	0	0	0	0	0	0
	3	1	1	1	0	0	1	1	1	1	1	0	1	1	0	1	1	1	1
	4	0	0	0	1	1	0	0	0	0	0	0	0	0	1	0	0	0	0
NABGRESM	0	0	0	0	0	0	0	0	0	1	0	1	0	0	0	0	0	1	0
	1	1	0	0	1	1	0	0	0	0	0	0	0	1	0	1	0	0	0
	2	0	1	1	0	0	1	1	1	0	1	0	1	0	1	0	1	0	1
NABGRESLM	1	0	0	1	1	1	0	0	0	0	1	0	1	0	1	0	0	0	0
	2	1	1	0	0	0	1	1	1	1	0	1	0	1	0	1	1	1	1
MPAb		0	0	0	0	0	0	0	1	0	1	1	1	0	0	1	0	0	0
FRSMVMAb		1	1	0	0	0	0	0	0	1	0	0	0	0	1	1	0	1	1
NCBMMVS	2	0	0	1	0	0	0	1	0	1	1	1	1	0	0	0	0	0	0
	≥3	1	1	0	1	1	1	0	1	0	0	0	0	1	1	1	1	1	1
FRSMVM		0	0	0	0	0	0	0	0	0	0	0	1	0	1	0	0	0	0

PR, presence of raphides; ET, expansion tissue (C = continuous, I = interrupted); cross-sectional shape of the midrib (Tri = triangular, Rou = rounded, Tru = truncated); SL, stomata at the same level of epidermis; BL, stomata below the level of the epidermis; ABSCM, accessory bundles surrounding completely the main vascular system of the midrib; FRMH, fibrous ring surrounding the vascular system of the midrib reaching the hypodermis; PCFRH, presence of at least one chlorenchyme layer between the fibrous ring and the hypodermis; EW, epicuticular waxes (T1 = inconspicuous deposits, T2 = deposit of horizontal plates, T3 = deposits of horizontal plates associated with hook-shaped filaments); NABMMVS, number of accessory bundles around the midrib main vascular system; ETE, expansion tissue stratification (2, 3 or 4 cell layers); NABGRESM, number of accessory bundles with greater reinforcement of sclerenchymatic sheath in midrib; NABGRESLM, number of accessory bundles with greater reinforcement of sclerenchymatic sheath in leaf margin; MPAb, midrib projected on the abaxial surface; FRSMVMAb, fibrous ring surrounding the main vascular system of the midrib projected towards the abaxial surface; NCBMMVS, number of collateral bundles in the midrib main vascular system; FRSMVM, fibrous ring wholly surrounding the main vascular cylinder in midrib.

**Table 2.** Useful anatomical characters for *Butia* taxonomy.

Characters	Jaccard index
Number of accessory bundles around the main vascular system of the midrib	0.71
Number of accessory bundles with greater reinforcement of sclerenchyma sheath in the midrib	0.62
Presence/absence of raphides	0.52
Shape of the midrib in transverse section	0.50
Characteristic of expansion tissue (continuous or interrupted)	0.50
Fibrous ring surrounding the main vascular system of the midrib projected towards the abaxial surface	0.50
Number of collateral bundles in the main vascular system of the midrib	0.47
Number of accessory bundles with greater reinforcement of sclerenchyma sheath at the leaf margin	0.47
Expansion tissue stratification	0.45
Midrib projected on the abaxial surface	0.42
Position of stomata in relation to ordinary epidermal cells	0.42
Distribution pattern of epicuticular waxes	0.39
Fibrous ring of the vascular system of the midrib reaching the hypodermis	0.37
Accessory bundles surrounding the main vascular system of the midrib completely	0.21
Fibrous ring surrounding the main vascular cylinder in the midrib completely	0.11

1 Presence of raphides.........................................................**2**
1′ Absence of raphides.........................................................**9**
2 Main vascular system of midrib composed of two collateral bundles............................................................**3**
2′ Main vascular system of midrib composed of three or more collateral bundles..................................................….**5**
3 Inconspicuous deposits of epicuticular wax.......................................................................................... ***B. leptospatha***
3′ Epicuticular wax deposited as horizontal plates..........**4**
4 Midrib round-shaped in transverse section…. ***B. capitata***
4′ Midrib triangular-shaped in transverse section..................................................................... ***B. marmorii***
5 Fibrous ring of the midrib connected to the hypodermis.................................................................................**6**
5′ Fibrous ring of the midrib not connected to the hypodermis.................................................................................**7**
6 Expansion tissue continuous......................... ***B. exospadix***
6′ Expansion tissue discontinuous................. ***B. pubispatha***
7 Fibrous ring projected on the midrib on the abaxial surface............................................................. ***B. campicola***
7′ Fibrous ring not projected on the midrib on the abaxial surface..................................................................................**8**
8 Accessory bundles surround by the fibrous ring of the midrib completely...................................... ***B. catarinensis***
8′ Accessory bundles surround the fibrous ring of the midrib partially................................................. ***B. microspadix***
9 Midrib protruding from the abaxial surface.................**10**
9′ Midrib not protruding from the abaxial surface..........**13**
10 Main vascular system of midrib composed of two collateral bundles.........................................................................**11**
10′ Main vascular system of midrib composed of three or more collateral bundles…..............................................**12**
11 Fibrous ring continuous..................... ***B. lepidotispatha***
11′ Fibrous ring discontinuous............. ***B. matogrossensis***
12 Fibrous ring of the midrib connected to the hypodermis............................................................ ***B. paraguayensis***
12′ Fibrous ring of the midrib not connected to the hypodermis............................................................ ***B. leiospatha***
13 Fibrous ring projected on the midrib on the abaxial surface…............................................................................. **14**
13′ Fibrous ring not projected on the midrib on the abaxial surface........................................................................... **17**
14 Expansion tissue four-layered...................................... **15**
14′ Expansion tissue three-layered…...............................**16**
15 Accessory bundles surround the fibrous ring of the midrib completely.............................................. ***B. odorata***
15′ Accessory bundles surround the fibrous ring of the midrib partially........................................................ ***B. yatay***
16 All accessory bundles of the midrib without a greater reinforcement of sclerenchymatic sheath......................................................... ***B. purpurascens***
16′ One accessory bundle of the midrib with greater reinforcement of sclerenchymatic sheath..... ***B. archeri***
17 Main vascular system of midrib composed of two collateral bundles.............................................. ***B. lallemantii***
17′ Main vascular system of midrib composed of three or more collateral bundles…............................ ***B. eriospatha***

**Figure 5.**
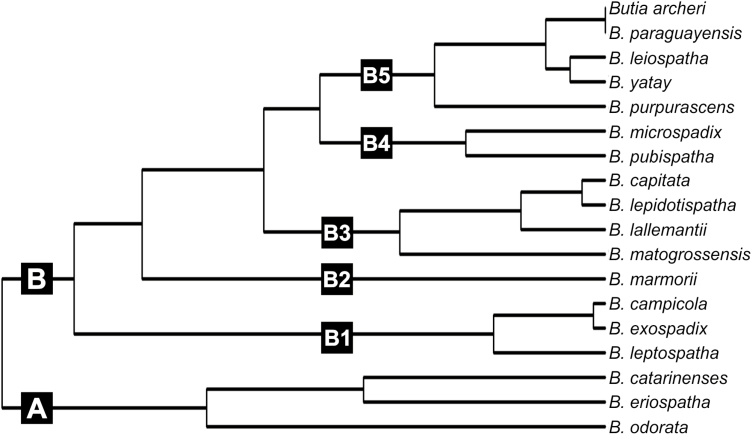
Dendrogram of similarity between *Butia* species based on leaf anatomy.

The resulting dendrogram identified two main groups, here called A and B (Fig. 5). Group A includes *B. odorata*, *B. eriospatha* and *B. catarinensis*, while group B includes the remaining 15 studied species of *Butia*. The characters that differentiate each group are four-layered expansion tissue (group A) and two- or three-layered expansion tissue (group B).

Within group B, five subgroups can be recognized. One of such subgroups is the group B1, composed of *B. exospadix*, *B. campicola* and *B. leptospatha* and share 10 characters, such as the presence of raphides and stomata at the same level of other epidermal cells. We found greater similarity between *B. exospadix* and *B. campicola*, corroborated by the presence of two accessory bundles of greater calibre within the midrib.

*Butia marmorii* alone composes group B2, which can be characterized by a more or less triangular midrib, 16–31 vascular bundles surrounding the midrib and two-layered expansion tissue. The group B3 is composed of *B. matogrossensis*, *B. lallemantii*, *B. lepidotispatha* and *B. capitata* that share some relevant characteristics such as stomata below the remaining epidermal cells, the vascular system of the midrib composed of two opposed bundles. Nevertheless, there is no exclusive character for this group.

Species included within group B4 (*B. pubispatha* and *B. microspadix*) share the highest number of anatomical characters (12), such as stomata at the same level of the remaining epidermal cells.

The group B5 is composed of five species lacking raphides (*B. paraguayensis*, *B*. *archeri*, *B. purpurascens*, *B. yatay* and *B. leiospatha*). Regarding species within group B5, our analysis could not discriminate between *B. paraguayensis* and *B. archeri* based on pinnae anatomy. This similarity is mainly due to the presence of 8 to 13 vascular bundles around the vascular system of the midrib and the presence of only one accessory bundle of greater calibre within the midrib, characteristics not shared with the other species of the group.

## Discussion

The broader sampling of *Butia* in the present study confirms the presence of many characters common to the genus, such as isobilateral mirrored mesophyll, amphistomatic leaves and vascular bundles with a sclerenchymatic sheath reinforcement connected to the hypodermis, as previously described (Tomlinson 1961; Tomlinson *et al.* 2011). Those authors also pointed out the structural divergence between the mesophyll of *Butia* and its sister group, *Jubaea* (*sensu*Meerow *et al.* 2009, 2015). Now that more species of *Butia* have been analysed both by molecular phylogenetic (Meerow *et al.* 2015) and structural analyses (this study), it is possible to propose the mirrored isolateral mesophyll of *Butia* not only as diagnostic of the genus but as well as a synapomorphy.

The species groups in the dendrogram reflect some of the already established taxonomic issues for the genus, morphological similarities or geographical proximity. Group A, represented by *B. catarinensis*, *B. odorata* and *B. eriospatha*, were never reported as problematic in distinction. Notwithstanding, these species naturally occur in relatively close areas, but the presence of raphides is restricted to *B. catarinensis*. Thus, anatomically, it is possible to distinguish them using a reliable character that is easy to obtain and recognize.

*Butia catarinensis* and *B. odorata* stand out for presenting accessory bundles surrounding the vascular system of the midrib, which do not occur in *B. eriospatha*. This character has been previously observed in *B. odorata* (Sant’Anna-Santos *et al.* 2015). *Butia odorata* has natural populations occurring near to *B. catarinensis* and disjunct by a narrow strip of seacoast (Noblick 2010). Although there are no reported difficulties to distinguish both species, the size of specimens and the shape and size of reproductive organs, the most commonly used characters to separate them, are known to be variable within the genus (Glassman 1970; Noblick 2010, 2014; Soares *et al.* 2014). Thus, for a reliable identification, the presence of raphides in *B. catarinensis* can be used to distinguish it from *B. odorata* and *B. eriospatha*.

Within group B, the first group of species is B1, which includes *B. exospadix*, *B. campicola* and *B. leptospatha* and belong to the same complex of species: the ‘grassy *Butia*’ (Noblick 2006). Morphologically, this group share many similarities, including the notorious graminoid size (Noblick 2006). However, the papyraceous minute peduncular bract of *B. leptospatha* (also observed in *B. marmorii*) notoriously distinguishes it from the other two species of group B1. Anatomically, *B. leptospatha* differs from *B. campicola* and *B. exospadix* by the number of vascular bundles within the midrib. Although *B. leptospatha* can be easily distinguished by its morphology, when devoid of inflorescences, the characters observed here assume great relevance. *Butia leptospatha* occurs remarkably near to the natural areas of occurrence of the other species within group B1. *Butia campicola* and *B. exospadix* morphologically also share many similarities and, according to Noblick (2010), ‘grassy *Butia*’ whose inflorescence is greater than the bract. The most obvious morphological difference lies in the size of the leaf rachis, much longer in *B. campicola* (Noblick 2010). Anatomically, the hook-shaped filaments, within this group, are unique to *B. campicola*, which may be useful to differentiate the latter from *B. exospadix* and *B. leptospatha*. This is especially relevant when there are doubts about the juvenility of the specimen, a factor that influences the size of the vegetative parts, as reported by Glassman (1970).

Group B2 is composed of *B. marmorii* only, differentiated from the other species by a peculiar leaf anatomy represented by three exclusive anatomical characters (EAC) (stratified two-layered expansion tissue, triangular midrib and 26–32 accessory bundles in the midrib). Those characters distinguish it from the other 17 species studied here. *Butia marmorii* is only known from a small area in Paraguay (Noblick 2010) and is one of the representatives of the grassy complex of *Butia*; Noblick (2010) pointed out a great morphological similarity between *B. marmorii* and *B. leptospatha*. Although there is no history of problems in distinguishing these species, the proximity of their areas of occurrence and make the EACs observed here are valuable. The remaining grassy complex, formed by *B. pubispatha* and *B. microspadix* (Noblick 2010), was included within group B4. *Butia pubispatha* was first collected and erroneously identified as *B. microspadix*, as reported by Noblick (2010). Morphologically, they can be distinguished by the indument; size of bract and number of rachillae. These characters can be influenced by environmental conditions (Metcalfe and Chalk 1979; Fahn and Cutler 1992), besides occasionally appear in certain species of the genus (Soares *et al.* 2014) and only become available during the reproductive period. Thus, it is expected that only experienced taxonomists have no difficulty in distinguishing between related species that occur in the same area, as already reported for these species (Noblick 2010). It thus becomes clear that data on the leaf anatomy, organ available throughout the year, contribute to distinguishing these two species. Therefore, anatomically these species can be distinguished by four characters, with an emphasis on the type of epicuticular wax (horizontal plates in *B. microspadix* and hook-shaped filaments in *B. pubispatha*), which, according to Barthlott (1981), is reliable for taxonomic purposes.

Group B3 includes *B. capitata*, *B. lepidotispatha*, *B. lallemantii* and *B. matogrossensis*. The last one can be easily confused with *B. capitata* when juvenile (Noblick 2010). However, anatomically, they can be discriminated based on nine characters, including the presence of raphides, which is restricted to *B. capitata*. *Butia capitata* is anatomically similar to *B. lallemantii*, but the latter is known from cespitose endemic specimens from the Rio Grande do Sul State, while *B. capitata*, in general, is represented by solitary individuals with apparent stipe from the Brazilian Central Plateau. There is, therefore, between these two species, not only a wide range of geographical but also morphological disjunction. Similar to *B. paraguayensis*, *B. capitata* seems to be a highly variable species (Glasmann 1970), and a broader taxonomic study is fundamental to better elucidate any questions regarding this group. It is worth mentioning that the anatomical characteristics observed in the present study in *B. capitata* (presence of raphides, midrib format, expansion tissue, accessory bundles and others) were similar to those of *B. odorata* and previously reported by Sant’Anna-Santos *et al.* (2015). Moreover, we analysed samples from other populations of *B. capitata*, indicating reliability in the use of these data presented here.

*Butia lepidotispatha* and *B. matogrossensis* consist of species that occur in different areas from Mato Grosso do Sul State (Noblick 2010). However, both were recently described, and it is likely that future collections will indicate a closer relationship between their populations. Morphologically, we find it difficult to distinguish *B. lepidotispatha* from *B. matogrossensis*. However, the lepidote indument of the stalk is indeed noticeable in *B. lepidotispatha*, and Noblick (2010) considers as a consistent and remarkable character for the species. Notwithstanding, according to Soares *et al.* (2014), this feature seems to be very variable, as it may occasionally appear in other species of the genus. Anatomically, although very similar, they also have a remarkable character that differentiates them: the presence of discontinuity points within the fibrous ring of the midrib of *B. matogrossensis*, an EAC of this species.

Within group B5, *B. paraguayensis* appears together with *B. archeri*, with only two different characters, which, in our analyses, were not enough to discriminate between these species. Nevertheless, *B. archeri* and *B. paraguayensis* are morphologically highly distinct and, to the best of our knowledge, do not occur sympatrically (although the southern limit of the populations of *B. archeri* being close to the northern limit of *B. paraguayensis*). There are no reports that these two are related species or even difficulty in their separation. As *B. paraguayensis* form a highly variable group, difficult to understand and probably form a complex and not a single species (Noblick 2014), a broader systematic study, using molecular tools and analysing several populations, is fundamental to elucidate better any questions relating to this group. We analysed samples from other populations of *B. archeri* too, indicating reliability in the use of these data presented here.

Within the other terminal of group B5, *B. yatay* and *B. leiospatha* are very similar. However, there are no reports of taxonomic problems and sympatric occurrence between them. *Butia leiospatha* has been anecdotally cited as a dwarf form of *B. archeri* (Plantarum Botanical Garden website, 2018), species also belonging to group B5. Presently, *B. leiospatha* is considered as a synonym of *B. capitata.* However, there are many anatomical differences between these species, including four characters with the highest value of Jaccard index. *Butia leiospatha* does not appear on the list of species of Noblick (2014), which shows that its status as a questionable species is still a consensus. Although it does not present any EAC, the anatomical data presented here support its segregation both from *B. archeri* and *B. capitata*. Due to the lack of a reliable type of *B. leiospatha*, it is premature to suggest its reinstatement as a valid species, especially because the specimen here analysed and previously identified as *B. leiospatha* could as well represent a new species. Thus, it is urgent to analyse this issue more thoroughly and review the status of this taxa.

*Butia paraguayensis* and *B. yatay* are difficult to understand due to the high morphological variability of their populations (Noblick 2014). *Butia yatay* is the only group B species that possesses a group A character (four-layered expansion tissue). Nonetheless, this exception can be explained by the close relationship between *B. odorata* and *B. yatay*, demonstrated by molecular phylogenetic analyses by Meerow *et al.* (2015). *Butia paraguayensis* has already been considered as a variety of *B. yatay* (Beccari 1916), but nowadays, instead, is a recognized taxon. Our data allow the differentiation between the former and the latter by eight characters, four of them with the highest values of Jaccard index. In Brazil, the geographical distribution of these species has a disjunction in the Rio Grande do Sul State: *B. paraguayensis* occurs more to the northwest of the state, while the natural populations of *B. yatay* are restricted to the southwest (Noblick 2010). However, in Argentina, *B. paraguayensis* specimens have been identified in areas of occurrence of *B. yatay*, which, according to Noblick (2014), might be a mistake. In Uruguay, the only existing population of *B. paraguayensis* was called by Noblick (2014) as an ‘odd disjunct population’, reaffirming the difficulty of identifying this species only by characters of the external morphology. Thus, the anatomical features identified here shall be considered for correct identification.

Still, regarding group B5, *B. purpurascens* appears on the base of the group within the dendrogram and, accordingly, is anatomically distinct from the remaining species. In the literature, there are no reports of difficulties in its identification, but its anatomical similarity to *B. paraguayensis*, *B. archeri* and *B. leiospatha* coincides with the type of environment in which they occur: sandy soil savannas (Noblick 2010).

Raphides were observed in seven of the studied species, not being a unifying character to *Butia*. However, this character is helpful to differentiate species that sometimes are confused. Among all the evaluated traits, raphides may show a few major advantages: convenience and low cost for obtaining the data, easy visualization and reliability for species distinction, having already been used successfully to discriminate between other morphologically similar *Butia* (Sant’Anna-Santos *et al.* 2015).

Even though it has been useful in distinguishing species from other genera, such as *Allagoptera* (Pinedo *et al.* 2016), *Oenocarpus* (Silva and Potiguara 2008; Tomlinson *et al.* 2011) and *Syagrus* (Noblick 2013), the leaf margin format showed intraspecific variation in part of the *Butia* species analysed here, reason that justified the exclusion of this character in the proposed key. Even when not variable within the same species, data such as epicuticular wax (Barthlott 1981) require more refined and costly methodologies. In this case, it is expected that the use of costly equipment may be an obstacle and limit the use of such data in taxonomic routine. As pointed out by Noblick (2014), considering the total number of *Butia* species described, solving taxonomic problems should be a priority in genus like *Butia*, morphologically very variable, with doubtful circumscription and composed of species complexes, new species and even species that have been described for decades and are still questionable.

Based on the external morphology, Noblick (2014) mentioned in his recent work: ‘I am not even going to pretend that I could really write a decent key to all of the *Butia*’. We believe that the key proposed here will, then, besides the two keys already proposed by Noblick (2010) and Soares *et al.* (2014), help expand our understanding of the genus and facilitate the identification of species. Keys including anatomical data are already being used in genus related to *Butia*, *Syagrus* for instance (Noblick 2013), and have shown great taxonomic potential and grounded the description of new species, as in *Allagoptera* (Martins *et al.* 2015; Pinedo *et al.* 2016). New anatomical data are especially valuable for extremely variable groups, like genera with species complex and many taxonomic issues. Also, such data have been used as an argument to raise or not a certain taxon, as observed in Noblick (2013, 2014) and Martins *et al.* (2015).

## Conclusion

The anatomical data proposed here enabled us to develop a purely anatomical key, in which specific identification is performed by a set of characters up to five. Also, two species can be identified by exclusive characters. The key proposed here has new and relevant characters for identification and can assist in solving taxonomic problems in *Butia*.

Previous studies on other groups of Arecaceae have successfully applied leaf characters to aid in species identification and to help to explain their evolutionary history (e.g. Noblick 2013). Thus, it is somewhat surprising the incongruence between the groups of species recovered by our analyses of the leaf anatomy and those depicted in the most recent molecular phylogenetic tree that included *Butia* (Meerow *et al.* 2015). These authors analysed seven species belonging to *Butia* and confirmed its monophyly and *Jubaea* as its sister clade. Notwithstanding, their analyses could not resolve the relationship between *Butia* species. In spite of being the first branch to diversificate within South America, *Jubaea* and *Butia* have diverged more recently than other genera belonging to Attaleinae (Meerow *et al.* 2015) and it is possible that the leaf diversity found in the present study is a reflex of this recent divergence. Given the dates of divergence estimated by the analyses of Meerow *et al.* (2015), fluctuations in the distribution of rainforest and seasonally dry climates in South America after the Andean uplift are likely responsible for many speciation events within *Butia*, prompting the extant leaf diversified anatomy, adapted to the different climatic conditions. Due to the limited sampling in previous analyses and the possibility of a recent diversification in *Butia*, it is necessary to carry out a new molecular phylogenetic analysis, including more species and using other molecular markers, more adequate to account for a higher diversification rate.

## Sources of Funding

This research was conducted with funds from Fundação de Amparo à Pesquisa do Estado de Minas Gerais (FAPEMIG—CRA APQ 01043/11) and the Pró-Reitoria de Pesquisa from the Universidade Federal de Minas Gerais (UFMG). E.L.P.N. was supported by Coordenação de Aperfeiçoamento de Pessoal de Nível Superior (CAPES—99999.000246/2015-08) and Alexander von Humboldt Foundation (BRA 1161587 HFSTCAPES-P).

## Contributions by the Authors

B.F.S.-S. and W.G.O.C.J. posed the central questions; S.A.S., B.F.S.-S. and D.M.T.F. performed the microscopic analysis; the authors analysed the data together; B.F.S.-S., S.A.S. and E.L.P.N. wrote the original manuscript; E.L.P.N. and B.F.S.-S. edited for content and provided guidance on structure and style.

## Conflict of Interest

None declared.
